# The nurse anesthetist perioperative dialog

**DOI:** 10.1186/s12912-020-00429-7

**Published:** 2020-05-08

**Authors:** Anna Abelsson, Annette Nygårdh

**Affiliations:** Department of Nursing Science, School of Health Sciences, Jönköping University, PO Box 1026, 551 11 Jönköping, Sweden

**Keywords:** Nurse anesthetist, Content analysis, Perioperative dialogue

## Abstract

**Background:**

In the perioperative dialogue, pre-, intra- and postoperatively, the patient shares their history. In the dialogue, the nurse anesthetist (NA) gets to witness the patient’s experiences and can alleviate the patients’ suffering while waiting for, or undergoing surgery. The aim of this study was to describe the nurse anesthetist’s experiences of the perioperative dialogue.

**Methods:**

The study had a qualitative design. Interviews were conducted with 12 NA and analyzed with interpretive content analysis. The methods were conducted in accordance with the COREQ guidelines.

**Results:**

In the result, three categories emerge: *A mutual meeting (the preoperative dialogue)* where the patient and the NA through contact create a relationship. The NA is present and listens to the patient, to give the patient confidence in the NA. In the category, *On the basis of the patient’s needs and wishes (the intraoperative dialogue),* the body language of the NA, as well as the ability to read the body language of the patient, is described as important. In the category, *To create a safe situation (the postoperative dialogue)* the NA ensures that the patient has knowledge of what has happened and of future care in order to restore the control to the patient.

**Conclusion:**

The patient is met as a person with their own needs and wishes. It includes both a physical and a mental meeting. In a genuine relationship, the NA can confirm and unreservedly talk with the patient. When the patients leave their body and life in the hands of the NA, they can help the patients to find their inherent powers, which allows for participation in their care. Understanding the patient is possible when entering in a genuine relationship with the patient and confirm the patient. The perioperative dialogue forms a safety for the patients in the operating environment.

## Background

The perioperative dialogue is a NA pre-, intra- and postoperative dialogue with the patient in connection with anesthesia or surgery [1]. The perioperative dialogue is defined as enabling the NA to protect the patient’s human dignity, alleviate suffering, and create a safe care environment. The patient can then get a feeling of well-being during their operative intervention. Through the perioperative dialogue, guidance for future planning of care and caring itself is been provided [1, 2].

In the *Preoperative* dialogue, the patient and the NA are meeting before the operation [2]. The meeting can take place in the patient’s ward or in the recovery room in the operating department [1]. The patient receives information and can ask questions which the NA can answer and reduce the patient’s anxiety [3]. The *Intra-operative* dialogue begins when the patient is received in the operating room, and they are then already familiar with their NA. The *postoperative* dialogue takes place in the recovery room in the operating department. In the postoperative dialogue, the patient has the opportunity to, after the operation, finish and evaluate the care process together with NA [2].

The perioperative dialogue allows the NA to spend time with the patient before and during the anesthesia. In the meeting, the NA and the patient can together plan the care throughout the perioperative process. In the perioperative dialogue, they both get to know each other [1] and establish trust [4]. Patients describe the perioperative dialogue as being able to share their history, which makes them feel safe when putting control of their bodies in NA’s hands [5]. In the dialogue, the NA can understand the patient’s experiences, which allows NA to alleviate the patients’ suffering while waiting for surgery [1]. According to Pulkkinen et al. [1] the patients express emotional feelings before surgery such as anxiety and fear, which may cause suffering.

The continuity in the perioperative dialogue ensures that the patient sees a familiar face inside the operating room. Continuity becomes an ethical act that is shown by the NA taking responsibility for the patient [2]. The responsibility entails that the NA is present for the patient. The patients experience a sense of safety to have the NA at their side throughout the perioperative care process [1, 6]. When the NA has the will to be present for the patient and his or her needs, it can empower the patient and give a sense of safety [4]. The will to exist for the sake of others is the ethos of professional caring. An ethos that is characterized by preserving human dignity [2].

For the NA the continuity of the dialogue is both a way of caring for their patient as well as a method of collecting data from the patient. Using the perioperative dialogue creates a common world for the patient and the NA [1]. In the common world, the possibility of establishing a caring relationship is created, a togetherness, allowing for dignified care of the patient [1, 7]. When this togetherness in the common world is not created, the patient can experience a lack of dialogue where the patient does not understand the information and therefore, may not be involved in decisions for their care [4]. The presence of the NA can confirm the patient and the caring relationship and togetherness [1]. To be confirmed is to experience oneself as a unique human being [4, 8, 9].

The perioperative dialogue is essential for patients to feel safe and that someone is close to them in a vulnerable and sometimes unknown care situation. The aim of this study was to describe the nurse anesthetist experiences of the perioperative dialogue.

## Methods

The study had a qualitative design. Interviews were conducted and analyzed with an interpretive content analysis inspired by Krippendorff [10]. The methods were conducted in accordance with the Consolidated criteria for reporting qualitative research (COREQ) guidelines [11].

### Participants

The participants in this study were NA, which is defined as a registered nurse with postgraduate education as a nurse anesthetist. A NA can independently induce, maintain, and end general anesthesia, with some support from the anesthesiologist [12]. In total, 12 NA participated; 7 males and 5 females, aged between 29 and 63 years (mean 52). The participants had an average of 13 years’ experience as NA, with a range of 1 to 30 years. All participants were informed about the study by their manager, and the interested individuals voluntarily contacted the researcher.

### Data collection

The interviews were conducted in private with one participant at a time at their place of work at an operating theatre department in the hospitals. All interviews started with the same open-ended question: *Would you like to describe your perioperative meeting with the patient?* This question was followed up by an in-depth question to get a more in-depth description, such as: *Can you give an example?* or *Can you describe what you mean?* (Additional file 1). The open-ended question was developed for the sole purpose of this study and pilot tested, and the two pilot interviews were included in the study. The interviews lasted approximately 20 min and were recorded and transcribed verbatim.

### Data analysis

The text consisting of transcribed interviews were analyzed using text-driven, interpretive content analysis by Krippendorff [10]. The interpretive analysis started with the familiarizing of the transcribed interviews. By reading with an open mind, it was possible to see beyond the already known and to reflect on the text that could be interpreted neutrally [10]. The text regarding the NA experiences of the perioperative dialogue was read repeatedly to reach an understanding of the substance of the data as a whole. After that, the text was read carefully to identify meaning units that represented experiences of the perioperative dialogue, in total 89 meaning units related to the preoperative dialogue, 66 meaning units related to the intraoperative dialogue and 17 meaning units related to the postoperative dialogue. The next step was to derive codes from the meaning units. Thereafter, the codes were abstracted and sorted into three categories based on similarities and differences; *A mutual meeting (preoperative dialogue)*, O*n the basis on the patient’s needs and wishes (intraoperative dialogue)* and *To create a safe situation (the postoperative dialogue*) (Table 1). When the analysis was done, a comparison was performed to identify how well the three categories synchronized in the analysis. The relevance of the results was finally verified by the correlation between the aim of the study and the categories [10].

**Table 1:** The process of how the three categories were generated

Meaning units	Codes	Categories
Conversation about the operation. Try to see and confirm the patient. Be present for the patient.	To confirm the patient	A mutual meeting (The preoperative dialogue)
For some, it is enough that you are calm and inform them, Others prefer it quiet, you work on and get the work done until they fall asleep.	Customizing information	A mutual meeting (The preoperative dialogue)
Being focused and present with the patient who is involved and determines for themself how it feels	Understanding the patient’s needs	On the basis of the patient’s needs and wishes (The intraoperative dialogue)
You feel that you have come close to a patient when you talk to them, and they relax. They even burst into tears because they feel safe. Then I feel that I have been successful.	Understanding the patient’s needs	On the basis of the patient’s needs and wishes (The intraoperative dialogue)
Perhaps the course of events is described very precisely so that they feel they know all the details. The experience is the same regardless of the type of surgery.	Give the control back to the patient	To create a safe situation (The postoperative dialogue)
It can be difficult to know sometimes that the patient has enough information to make it at home.	To ensure that the patient has knowledge	To create a safe situation (The postoperative dialogue)

## Results

The results are presented in three categories: *A mutual meeting (the preoperative dialogue), On the basis of the patient’s needs and wishes (the intraoperative dialogue)* and *To create a safe situation (the postoperative dialogue*) based on the NA’s stories from their meetings with the patients.

### A mutual meeting (the preoperative dialog)

The NA described how, in the first meeting, they wanted to create personal contact with the patient. This meeting laid the foundation for how the relationship would continue. Therefore, in the first meeting with the patient, the NA describes how they made a little extra effort. To greet the patient and to introduce themselves with name and title was described as important. The introduction gave the patient the feeling of safety and confidence in the meeting and a relationship. A relaxed and pleasant meeting enabled a relationship while, in contrast, a tense meeting resulted in a substantial amount of work for the NA to get a working relationship with the patient.

Through the experience of having met many patients with different needs, the NA had the ability to reach the patient in the initial meeting. This ability also made it possible for the NA to reach the patient in the short amount of time given for the first meeting. Because the meetings were short, the NA had to compress the content of the meeting. Compressing was possible by briefing the information provided and quickly building a perception of the patient. However, regardless of how compressed the meeting was, the NA described that they always strived for good quality.

*When it comes to being able to reach people, you bring out everything you learned as well as sharpen the discussion to get as good a meeting as possible in a short amount of time.*

When a relationship between the patient and the NA had been established, the patients seemed to experience confidence in the NA. Confidence was based on that the NA had an honest interest in the patient. A mutual contact resulted in a natural meeting. The NA described that the patient was involved and seen as a person who was important in the meeting. The patient was able to talk about themselves, and the NA was present and took the time to listen. The NA describe the importance of being both physically and mentally present in the meeting with the patient. Not being present meant having their thoughts somewhere else, which patients felt and resulted in a meeting where only one party was participating. When the NA was stressed, and in a hurry, there was no reciprocity because the NA described how they did not wholeheartedly participate in the conversation and the meeting with the patient.

*The patient feels that you are stressed. Sometimes you are in a hurry and maybe thinking about something else. The patients can feel it.*

It was evident how important it was to focus on the conversation with the patient and let the patient decide the pace of the conversation. In the conversation, the NA created a picture of each patient and the needs of the individual patient. It was described how all patients were individuals with different needs who would be treated in different ways. It was the ability of the NA to read the individual patient and their needs that determined what information the patient was ready for during the course of the meeting.

*You cannot run the same concept on each patient, but you are considering whether the patient is taking in the information and what information does the patient need to have.*

In the conversation with the patient, the NA described how they could get a feeling for the patient based on the patient’s personality, which helped the NA to provide an adapted treatment. With questions, the NA could understand what mood the patient was in. Being responsive to the answers helped the NA to respond to the patient.

*One can quickly see if it is a person who looks terrified or a person who seems safe, sad, or angry. You try to find a good way to approach the patient. You have to be perceptive.*

Being perceptive helped the NA in knowing how much information the NA should share with the patient. The NA described how they could sense which patients wanted information and who did not want to know anything about anesthesia. By informing patients about the essentials and then listening to the patient, the NA decided to what degree they shared information.

*Some patients want short answers, and some want to know everything to gain control. My answers are adapted to the patient. Some patients just want to be cared for and not have information at all. So, this sorts itself out.*

Informing the patient about what was going to happen in the operating room was considered to soothe the patient. When patients had an informed picture of their anesthesia, the NA felt that the patient was prepared. Lack of information before anesthesia was described as causing fear in the patient. The NA described how they wanted to avert the patient’s fear of anesthesia. By sitting down with the scared patient, the meeting could focus on what worried the patient. By asking direct questions about patients’ fears, the NA could answer questions and sort out misunderstandings or incorrect knowledge.

*Some patients say directly- I’m really scared. I think this is very uncomfortable and I’m very nervous. Then I take the time to sit down and talk a little longer. Then I can ask the patient what they are afraid of and often easily answer them.*

Conversing with the patient also meant observing a patient’s body language. It was revealed how the need for information for nonverbal patients was more difficult to identify. The patients who were nonverbal could instead be observed by their body language that showed if they were feeling safe or scared. An assessment could also be made via vital parameters such as heart rate and blood pressure.

The fear of anesthesia was described as being afraid of losing control of their body and life. A common fear described by the NA was that the patient would not wake up after the anesthesia. It was, therefore, described how important it was to create a sense of safety in a situation where the patients experienced being unsafe and frightened.

The NA described how a sincere interest in the patient showed the patients that they could dare to put the responsibility of their breathing and their lives in the hands of the NA. When the NA was there for the patient and replied to the questions and informed the patient, the NA and the patient came closer together, creating trust in the relationship.

*We can explain this in 10 s, instead of the patient getting into the operating room with the thought of -will I wake up? I talk to the patient about this, and then it feels like I gain their trust.*

A NA acting professional in their work gave the patient a sense of safety, and the NA’s calmness was transferred to the patient. Patients also gave signs of gaining increased confidence in the NA when the NA talked about their experience and knowledge of anesthesia and prior involvement in surgeries. Patients then could understand that anesthesia was often not difficult, and rather a routine task performed daily. The NA answered the patients’ questions on similar surgeries based on previous experiences.

*Patients can be very focused on whether the surgery they are undergoing tend to fail or if there are typical problems during the surgery. And I can answer that easily as I have been in many similar surgeries.*

The NA’s experience was also needed when patients expressed personal experiences of previous anesthesia. Previous negative experience required a greater effort from the NA to get the patient to feel safe and calm. It was more difficult to create confidence in a patient who was afraid because of past experiences.

When a patient lacked important information from the operating surgeon, the NA described that the patients were not able to create an understanding of what was going to happen. The patients had a hard time feeling safe. The NA then described how they arranged a meeting with the surgeon to inform the patient before the surgery.

To meet the patient and establish a working relationship where the patient trusted the NA and could feel safe with the NA was described as the most important aspect of the preoperative conversation. The goal was that the patient would be given a good experience and not be afraid or anxious about the anesthesia.

### On the basis of the patient’s needs and wishes (the intraoperative dialogue)

When the patient entered the operating room, the NA wanted the patient to be treated and feel like a human being. The opposite was when the human aspect was forgotten, and the focus was solely on the surgery. The NA described how the staff in the operating room could greet and confirm the patient with a nod and eye contact. The staff of the operating room could confirm the patient by seeing the patient, not necessarily by approaching the patient. All staff in the room were not considered to have to introduce themselves and shake hands with the patient. Even as the patient was to be confirmed, it was considered too intrusive when too many people leaned over the bed where the patient was. To greet all the staff could also be described as stressful and something the patient could not always be able to do.

*The patient is not just a person lying in bed. If all of us storming in and there are 20 of us leaning over the patient. It is really quite threatening. It is important that you have the time or try to think about seeing the person lying there. Becoming blind to the person is easy.*

The NA described how they were constantly aware that the patient heard and saw much of what happened inside the operating room. To handle the patient’s fears of the anesthesia, the NA told the patient about what would happen during the anesthesia and showed the devices that were surrounding the patient.

When NA was aware that a patient was in extraordinary need of feeling safe, much like as a patient with mental retardation or a child, the staff in the operating room were asked to lower their tempo and work quieter. This gave the patient peace and quiet without disturbing or frightening them.

It was described how the NA, through different strategies, came closer to the patient in the operating room. NA could then focus on the patient and interpret the wishes of the patient. It meant to approach the patient in a way that was adapted to the patient’s personality and needs. The patient was listened to and was involved in the impending anesthesia.

*To listen to what the patient says and take them seriously. Not to over-rule the patient without reason. I believe that it is a question of listening and of having them understanding the reasoning. Then it will be a good anesthesia.*

Interpreting the needs of patients resulted in the NA being able to identify the patients who, during the anesthesia, wanted to talk about the anesthesia, those who wanted to talk about other things or those who did not want to talk at all. Adapting based on the patient’s needs created a calmness of the patient. The NAs described how they could get close to the patient in the conversation. It created a feeling of safety, and the patients relaxed when the NA encouraged the patient to speak up or ask if they had any questions during the anesthesia.

To provide patients safety in the operating room, the NA also used eye contact, body language, and touch. An anxious patient was spoken to while the NA looked the patient in the eyes and had physical contact. Consolation could be given with a pat on the cheek or by putting a hand on the patient’s shoulder. Through a body language that invited the patient to participate and by showing a genuine involvement in the patient, the NA could create a safe environment and situation in the operating room. The patient seeing and recognizing the NA at the head of the bed could sometimes replace words that did not need to be said.

*They should feel that you are still present and not talking too much as it can make some patients feeling stressed. I try to adapt to the patient, both what the patient says and what they radiate purely physically.*

The anesthesia was individualized and adapted to the patient’s preferences. Some patients wanted to fall asleep immediately and experienced information as a burden. Some patients could be described as nervous and stiff and angry. Regardless of how the patient’s fear of anesthesia expressed itself, the NA saw it as an important task to create a safe environment for the patient. The NA told the patient that they would be with the patient when they fell asleep while sleeping, and when they woke up. The aim was to get the patient to dare to release control and hand over responsibility for their breathing to the NA.

*I want a patient to feel confident that I will anesthesia them and that it is I who will guard them while they are asleep. Then I have a calm patient when they fall asleep and a calm patient when they wake up.; they even if they do not remember me specifically, they will have a positive experience of the anesthesia.*

The NA described that with experience and confidence in their profession, the NA could involve some of their duties to the patient. Patients who wanted control were given control to create calmer sedation. For example, patients who wanted could hold their masks during anesthesia.

For those patients who wanted information, the NA shared the information step by step to make it understandable. The information could be to describe how the surgery would be performed, to speak about the equipment inside the operating room or the effect of different medicines on the body. However, it was revealed how information sometimes risked creating confusion for the patient as opposed to helping the patient. The NA was, therefore, careful to always adapt the information based on the patient and his or her ability to digest the information.

Patients who wished were allowed to be involved in the care. If the patients wanted and could cope, in surgeries where this was a choice, they could decide whether they wanted to have sedation or how much sedation they wanted. The NA let the patients choose and, at the same time, informed them that the patient could always change their decision during the surgery. If they changed their mind, they got sedated afterward. Making decisions could, however, be perceived as a burden during the anesthesia, and in those cases, the patient often chose to transfer decisions to the NA. Patients showing signs of panic could be persuaded to receive some anesthesia to help them cope with the surgery. A slumber was considered more humane than the fear during a surgery.

The environment around the patient was also adapted according to their wishes. Pillows, blankets, and sleeping postures could be determined by the patient as long as it worked purely surgically. Offering music in headphones allowed patients to be awake but not to hear what happened in the room.

*You try to engage the patients and get them to participate, they should be able to influence. If the patient is in focus, it prevents them from being made into objects.*

An awake patient meant that the NA could communicate with the patient during the anesthesia. This allowed the NA to feel the patient’s need for closeness, safety, or the sedation that the patient needed. The NA was able to interpret, understand, and accepted the different wishes of the patient. If the patient wanted to talk, they had a conversation, but if the patient wanted to be silent or slumber, the NA respected it. However, they always informed and showed the patient that they were at the head of the patient during the entire surgery, regardless of whether they were speaking or not.

### To create a safe situation (the postoperative dialogue)

When NA had established a preoperative relationship with the patient and the patient had been able to release control to the NA, the NA described how the patient could wake up in peace and safety. The NA strengthened the patient’s safety by telling the patient that the procedure was completed. It was also described as important to quickly tell the patient that the surgery was over so that patients would not think they had woken up during surgery.

*As soon as they wake up, I try to tell them that the operation is over and everything has gone well. I feel they shouldn’t think they wake up during the surgery when they see all the lights.*

The NA described how the postoperative dialogue was based on the type of surgery or examination the patient had gone through. During cancer examinations of mainly young people, the postoperative conversation was a difficult conversation as patients expressed sadness and fear.

*For her, it’s a disaster. After they wake up, they cry rivers out there.*

The NA also described how they experienced the difficult postoperative conversation when an operation or examination revealed cancer.

*I tell the patient that the surgery has gone well, which I believe we should always do, even if it has not gone well. One tries to maintain the patient’s sense of safety.*

The NA could also choose not to say that the surgery had gone well, rather express oneself in a way that did not focus on what the surgery had shown, and instead, the anesthetic had ended.

*One focus on some other issues, I tend to be quite relaxed and say “Is it not nice that it’s over”.*

After deciding how much information the patient wanted prior to the surgery, the NA described that the patient expressed a feeling of being satisfied iwhether they were speakingn the postoperative dialogue. Postoperatively, the NA was able to tell the patient how the surgery had been done and that the patient had been given various medications. With information preoperatively, the NA described how patients postoperatively expressed that they felt that the surgery had gone well.

*They feel that it went well, it was not as bad as they thought and that they had been safe.*

In the postoperative dialogue, the NA was able to have conversations with patients about possible future surgeries and how the patient felt about it. A good contact between the patient and the NA was experienced as a prerequisite for patients to feel safe in future surgeries.

The postoperative conversation also included for NA to make sure that the patient had enough information to get by at home after the operation. That the patient knew where to turn for help in case of fever or bleeding.

*They should know what to do if they get an infection, if the dressing bleeds through, if they are in pain, if they can’t urinate when they get home.*

The NA was aware that the amount of information could be too much for the patient to handle postoperatively. They, therefore, used different options, such as involving a relative in the postoperative conversation or writing down certain information.

## Discussion

The ethos of the perioperative dialogue is, according to Lindwall et al. [13], dignity, which is embedded in the NA’s duty to think and act in a sensitive manner [13]. In the results, the NA describes how they see the patient as a human being. In an authentic meeting, the patient is not an object. The patient is a subject involved in creating their own “I”, as well as the NA participating in creating their own “you” for the patient. Buber [14] says that when we say “you” to another person, we ascend into a relationship with the other man. A relationship characterized by the “I” meets “You” and “You” meets “I” [14].

The meeting that takes place between the patient and the NA is unbiased, open-minded, and unprejudiced. Between You and I, there is no prior knowledge and no preconceived views. The true encounter resembles a symbiosis between the patient and the NA. You and I enter into an interpersonal room that has no claim to the other. The ego as a person continues to exist with its integrity and measure of the private sphere [14]. In this interpersonal room, the patient and the NA become one in both mental and physical existence. They both meet and see each other as people, and they speak. With the help of the language, Buber [14] means that we can acknowledge each other and the being as a human. With the language and respect for whether the patient wants to speak or be quiet, the NA can be close to the patient in the perioperative dialogue. When the patient wants to tell her stories, they are given time to do so. Because when the patient experiences a permission to talk about their sick body, the suffering can be alleviated [15].

Understanding the patient is possible when the NA enters a genuine relationship with the patient. In a relationship with the patient, the NA can confirm the patient. Confirmation is a basic human need and is based on the authenticity of the interpersonal [14]. Authenticity is an unconstrained and unreserved conversation where the patient and the NA turn to each other in truth. Then the genuine conversation can take place. The perioperative dialogue creates safety for the patients in the operating environment [1]. The safety that the perioperative dialogue creates is the responsibility of the NA that, according to Lévinas [16] is based on the NA’s ethical values.

For the patient in perioperative care, it means to hand over his life in other people’s hands, as also described by Pulkkinen et al. [1]. NA needs to find the inherent forces of the patient and lead the patient through the path that the patient wants to go. Buber [14] describes the person who has the ability to attract and use these forces, both in themselves and in the others. This person also dares to rely on these forces [14].

### Limitations

A limitation in this study may be that the NA who has an interest in the perioperative dialogue chooses to participate in the study. If this was the case, it might have affected the result. The interviews have had the NA’s experiences of the perioperative dialogue in focus. This means that it is the NA’s description, contrary to the patient’s own description, of the perioperative dialogue, which is a limitation.

## Conclusion

In the perioperative dialogue, the patient is met as a person with their own needs and wishes. The meeting includes both a physical and a mental meeting. In a genuine relationship, the NA can confirm and unreservedly speak with the patient. When the patient leaves his body in another’s hands, the NA helps the patient to find his or her inherent powers, which allows for participation in their own care.

### Relevance for clinical practice

From the findings of this study, we can make some conclusions with relevance for clinical practice. In an authentic perioperative meeting, the patient is seen as a subject involved in creating their own person and identity. The true meeting resembles a symbiosis between the patient and the NA and is unbiased, open-minded, and unprejudiced. In an interpersonal room, the patient and the NA meet and see each other and, with the help of the language, can acknowledge each other. Understanding the patient is possible when the NA enters a genuine relationship with the patient and confirm the patient. The perioperative dialogue creates a needed safety for the patients in the operating environment. NA needs to find the inherent forces of the patient and lead the patient through the path that the patient wants to go when handing over one’s life in the NA’s hands. The perioperative dialogue can be used to protect the patient’s human dignity by strengthening the staff at their core of care. Further research would be of value to clarify the importance of the perioperative dialogue as a guiding principle for both staff and patients.

## Supplementary information

**Additional file 1 **Interview Guide. Interview question: *Would you like to describe your perioperative meeting with the patient?*.Follow up questions: *Can you give an example? Can you describe what you mean?.*

## Data Availability

The datasets generated and/or analyzed during the current study are not publicly available for preserving anonymity and integrity of the participants but are available from the corresponding author on reasonable request.

## Abbreviation

NA: Nurse anesthetist
