# Supplementary material for: The nurse anesthetist perioperative dialog
Source: BMC Nurs. 2020 May 8;19:37. doi: 10.1186/s12912-020-00429-7 (PMC7206746; doi:10.1186/s12912-020-00429-7)
Supplement: Supplementary file 1 — Additional file 1 Interview Guide. Interview question: Would you like to describe your perioperative meeting with the patient?.Follow up questions: Can you give an example? Can you describe what you mean?. [file 12912_2020_429_MOESM1_ESM.docx]

Additional file 1
Interview Guide

Interview question: *Would you like to describe your perioperative meeting with the patient?*

Follow up questions:
*Can you give an example?
Can you describe what you mean?*
